# Segmental variation of myocardial deformation in patients with suspected ischemic heart disease

**DOI:** 10.1186/1532-429X-14-S1-P2

**Published:** 2012-02-01

**Authors:** Henrik Haraldsson, Johan Kihlberg, Jan E Engvall, Tino Ebbers

**Affiliations:** 1 Department of Medical and Health Sciences, Linköping University, Linköping, Sweden; 2 Center for Medical Image Science and Visualization, Linköping University, Linköping, Sweden

## Summary

The aim of this study is to investigate if the use of individual, per segment reference values for cardiac strain allows for improved discrimination of pathological deformation. In a cohort of patients with a high likelihood of ischemic heart disease, preliminary results within the subgroup of patients without pathological findings on cardiac MRI suggest a spatial dependency of strain.

## Background

In addition to perfusion and viability, systolic myocardial function constitutes one of the cornerstones in the evaluation of ischemic patients. Technical achievements have improved the accuracy of the quantitative assessment of myocardial function, allowing for better discrimination between healthy and diseased myocardium. These advances cannot be fully exploited without knowledge of the spatial dependency and individual variance of deformation in health and disease.

## Methods

In an ongoing study, a large cohort of patients with moderate chest pain and a high likelihood of coronary artery disease undergo cardiac MRI including cine loops for wall motion, perfusion at rest and during adenosine stress, acquisition of myocardial deformation with displacement encoding with stimulated echo (DENSE), and late gadolinium enhancement to demonstrate scar tissue.

In this preliminary study, patient demonstrating maintained perfusion, normal wall motion, and no signs of scar at late gadolinium enhancement were selected for further evaluation.

Regional deformation acquired with DENSE was evaluated in terms of radial and circumferential strains. All measurements were reported according to the seventeen segment model of the AHA. The apical cap was excluded from the quantification of regional deformation.

## Results

**Figure 1:**
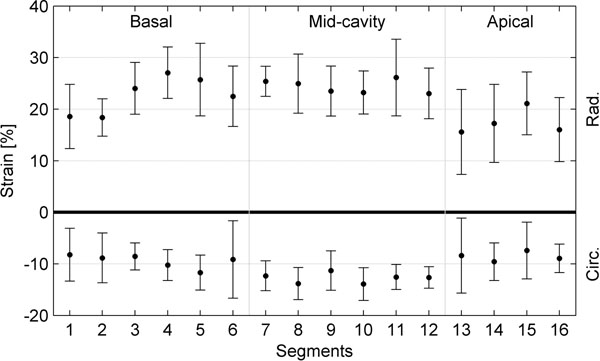
Radial and circumferential strain in the myocardial segments.

Preliminary results for radial and circumferential strain for the 11 patients without pathological findings are presented in the Figure 1. The figure demonstrates the mean and standard deviation of the strain for the individual myocardial segments. Strain in the basal segments appears to be more spatially dependent than strain in the mid-cavity segments. The strain in the apical segments showed a larger variation between the subjects.

## Conclusions

Preliminary results suggest a spatial dependency in deformation, larger than the fairly low standard deviation obtained using DENSE MRI. Hence, taking natural segmental variation of myocardial deformation into account might allow for better discrimination between normal and impaired myocardial deformation.

## Funding

County Council of Östergötland, Heart Foundation, Swedish Heart-Lung Foundation, and Swedish Research Council.

